# Emerging perspectives in the management of IgA nephropathy: a comprehensive review

**DOI:** 10.1097/j.pbj.0000000000000264

**Published:** 2024-11-14

**Authors:** Ana Marta Gomes, Bruno Schau, Ana Farinha

**Affiliations:** a Nephrology Department, Centro Hospitalar Vila Nova de Gaia/Espinho, Vila Nova de Gaia, Portugal; b UMIB/ICBAS—Unit for Multidisciplinary Research in Biomedicine/Instituto de Ciências Biomédicas Abel Salazar, Porto, Portugal; c CSL Vifor, Lisboa, Portugal; d Nephrology Department, Hospital de Vila Franca de Xira, Vila Franca de Xira, Portugal

**Keywords:** budesonide, complement system, ESKD, glomerular diseases, glucocorticoids, IgA nephropathy, proteinuria, RAAS inhibitors, SGLT2 inhibitors, sparsentan

## Abstract

IgA nephropathy (IgAN) is the most prevalent form of primary glomerulonephritis worldwide and a leading cause of chronic kidney disease and renal failure. This disorder is characterized by the deposition of immune complexes containing galactose-deficient forms of IgA and complement C3 in the glomeruli. Until now, disease management relied mainly on optimized supportive care. Systemic corticosteroid therapy is proposed for patients at high risk of disease progression, but the effectiveness and safety of this approach are under debate. A significant proportion of patients do not respond to current therapies and require kidney replacement therapy at a young age, with substantial costs and impact on quality of life. Recently, there have been multiple joint efforts to improve the understanding of IgAN pathophysiology. International collaborations resulted in multiple ongoing clinical trials that are providing new insights toward innovative therapeutic options such as SGLT2 inhibitors, dual endothelin and angiotensin receptor blockers, targeted-release budesonide, B-cell proliferation and differentiation inhibitors, and complement system blockers. Based on this new evidence, revision of the guidelines to manage IgAN is expected to occur in the near future. In addition to the novelty in therapeutic agents, there is also a growing interest in new noninvasive biomarkers for IgAN screening, risk stratification to monitor the course of the disease, and the response to treatment. In this review, we discuss current knowledge on the pathophysiology of IgAN, disease management, and emerging advances in clinical translation of IgAN research.

## Introduction

IgA nephropathy (IgAN) stands out as the most prevalent primary glomerular disease worldwide, accounting for a significant share of the global burden associated with end-stage kidney disease (ESKD).^1,2^ IgAN was first described more than 50 years ago. Its clinical heterogeneity presentation was recognized, but until recent years, treatment was not effective in delaying the progression of the disease. Currently, after years of stagnation, substantial several novel therapeutic approaches for the management of IgAN are emerging, mainly due to an increased recognition of the pathophysiologic mechanisms of the disease. In this review, we describe up-to-date information on the epidemiology, risk factors, clinical features, pathology, and innovative treatment strategies of IgAN.

## Epidemiology

IgAN is most common in young adults and children. In Western populations, adults are commonly diagnosed around the age of 40–45 years.^2^ Nevertheless, in countries with proactive urine screening programs, such as Japan, diagnoses tend to occur at earlier ages.^3^

Data regarding the prevalence of IgAN have shown a remarkable variation across different geographical areas: of all kidney biopsy diagnoses, Asia has the highest prevalence (30–60%), Europe exhibits a moderate prevalence (20–30%), and Africa has the lowest prevalence with less than 5%.^4,5^ A recent systematic review of 16 international studies shows that IgAN incidence varies from 0.06 in South Africa to 4.2 cases per 100,000 per year in Japan.^6^ In Europe and North America, IgAN reportedly has a moderate annual incidence of 2.5 cases per 100,000.^7^ Another review focused on European studies of national kidney biopsy registry data estimated an annual IgAN incidence of 0.76 per 100,000 in patients of all ages and a point prevalence (the annual IgAN incidence multiplied by the estimated duration of disease) of 2.53 per 10,000.^8^

In Portugal, according to the national renal biopsy registry, IgAN was the most common diagnosis between 2018 and 2022 (13.4%).^9^ In 2022, there were 112 new IgAN diagnoses in a total of 411 renal biopsies performed.^9^ 69% of these patients were male, and the mean age of diagnosis was 45.1 years.^9^ At the time of the renal biopsy/IgA nephropathy diagnosis, patients exhibited creatinine values of 2.24 mg/dl and proteinuria of 2.6 g/day.^9^ The main reason to undergo a renal biopsy was asymptomatic urinary abnormalities (27%).

It is important to consider, however, that these reports vary depending on the primary source of data. Data obtained from biopsy registries are prone to undervalue the incidence of IgAN because of the lack of inclusion of patients with mild disease, who often do not undergo biopsy. In fact, indications for kidney biopsy differ according to the country, leading to variations in definitive diagnosis of IgAN.^1,10^ For example, in Africa, only 0.8% of biopsy-proven primary glomerular disease has been reported, reflecting low accessibility to kidney biopsy and higher clinical thresholds to use costly examinations.^11^ In addition, geographical differences in the incidence and prevalence of IgAN could also be due to the fact that mass urine screening programs are systematically performed in several Asian countries, allowing detection of microscopic hematuria and/or mild proteinuria and early referral to a nephrologist.^10^

Despite these limitations in accessing the exact number of diagnosed patients across populations, diversity in the genetic background is also known to play a major role. Recently, a genome-wide association study (GWAS) with 17 international cohorts identified 16 new loci and defined 30 genome-wide significant risk loci that justify 11% of disease risk.^12^

## Clinical presentation and disease evolution

The clinical course of IgAN can present a broad range of manifestations. The 2 most common clinical presentations of IgAN are asymptomatic hematoproteinuria and chronic kidney disease (CKD) that are detected in routine urine and blood analysis.^7^ Other common presentations can be the appearance of macroscopic hematuria 1–2 days after the onset of a respiratory infectious synpharyngitic hematuria.^4,10^

Microscopic hematuria with minimal proteinuria was regarded as having a favorable prognosis.^13^ However, recent findings showed that the amount and progressive increase of proteinuria have a significant impact on long-term renal outcomes.^13^ CKD is also a common phenotype in multiple cohort studies,^7^ reflecting the late referral of many patients. In fact, most diagnoses are established in patients with proteinuria higher than 1–2 g/day and impaired renal function.^10^

Although less frequent, synpharyngitic hematuria is a classic clinical manifestation of IgAN, and due to the alarming nature of the symptom, it encourages patients to seek immediate medical care. It has been associated with a short-term favorable prognosis. Nevertheless, the development of persistent proteinuria is linked to the progression of the disease.^14^ There are also a few rare IgAN clinical manifestations that will not be covered within the scope of this review, such as rapidly progressive CKD and minimal change disease.

Despite the variable course of the disease, most patients undergo an inexorable decline in renal function and 10–60% reach ESKD 10–20 years after the diagnosis, mainly in younger patients.^15,16^ To prevent the progression of the disease to kidney failure, it is crucial that IgAN is detected and managed as early as possible. For this reason, the current approach to patient care needs to be re-evaluated through, for example, lowering the threshold for biopsy before irreversible damage occurs.^16^ Currently, proteinuria levels below 0.88 g/g (100 mg/mmol) have been indicated as a target to assess treatment response and inclusion of patients in clinical trials.^16^ In line with this, and owing to the asymptomatic onset of IgAN in many cases, routine urine analysis in young and active patients could be an interesting approach for early diagnosis and therapeutic decisions. Importantly, a significant number of patients who eventually require kidney replacement therapy are young adults (24–54 years old), with 22% of them younger than 30 years.^17^ This would have a key impact also from an economic perspective by reducing the need for this type of expensive therapy and the associated financial burden in health care.^10^

## Pathogenesis

IgA is a class of immunoglobulins present in large amounts in mucosal secretions. They provide immunity against local infections, tolerance to environmental pathogens, and sustain the microbiome.

### Multihit model of IgAN pathogenesis

**Figure 1.**
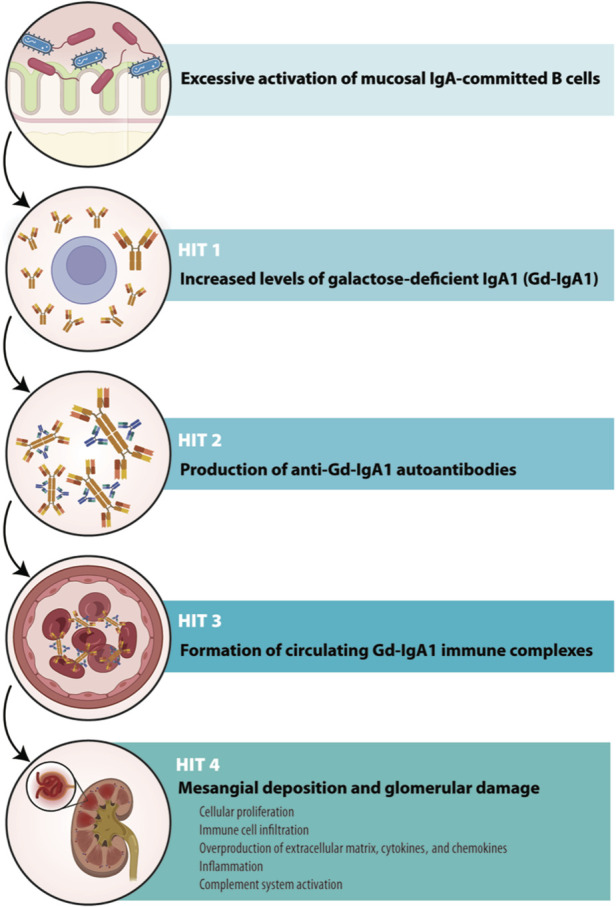
Pathogenetic multihit model of IgA nephropathy. Defective mucosal immune responses and antigen processing, among other upstream factors, exert a direct influence on 1 or more pathogenetic pathways in IgA nephropathy. Specifically, there is an increase in the circulation of IgA1 with galactose-deficient O-glycans (Gd-IgA1) in patients with IgA nephropathy (Hit 1). These Gd-IgA1 molecules are identified as autoantigens by antiglycan autoantibodies (anti-Gd-IgA1 autoantibodies; Hit 2), leading to the formation of nephritogenic immune complexes (Hit 3). Some of these complexes deposit in the kidney, triggering the activation of mesangial cells (Hit 4). Subsequently, mesangial cells undergo proliferation and excessive production of components in the extracellular matrix, cytokines, and chemokines. Some of these cytokines, in turn, contribute to podocyte injury, ultimately inducing proteinuria. The progression of these pathogenetic steps is likely influenced by various environmental and genetic factors.

Although the pathogenesis of IgAN is still incompletely understood, there is a widely accepted multihit model that gathers multiple factors that jointly trigger the development and progression of the disease (Fig. 1; Box 1). These include genetic predisposition, environmental triggers, and abnormal galactosylation of IgA1 and formation of IgA-containing immune complexes.^18^ According to this theoretical framework, the first event in the development of IgAN is the presence of elevated levels of circulatory galactose-deficient IgA1 (Gd-IgA1) (Hit 1), which leads to the production of autoantibodies of IgG, IgA, or IgM isotypes (Hit 2). The recognition of Gd-IgA1 by these antibodies leads to the formation of circulating immune complexes (Hit 3). These immune complexes eventually deposit in the glomerular mesangium, activate mesangial cells, and induce renal injury (Hit 4).^7,19^

Box 1. **Multihit model of IgAN pathogenesis.**

**Table:** 

Hit 1	Both IgA1 and Gd-IgA1 molecules are typically produced by mucosal IgA-committed B cells. Pathogenic alterations of IgA1 involve reduced galactosylation of O-linked N-acetylgalactosamine (GalNac) residues, with or without altered sialylation in the hinge region.^18^ Currently, it is thought that the gut microbiome or mucosal pathogens of the gastrointestinal tract can stimulate innate immune cells through Toll-like receptors, leading to overactivation of mucosal B cells. This originates an excessive production of Gd-IgA1 that enters circulation from mucosal sites or from systemic sources, due to the miss-homing of mucosal B cells^4,18^
Hit 2	The GalNac regions of Gd-IgA1, when exposed, can represent an epitope recognized by specific antiglycan antibodies (IgA, IgG, or IgM). Formation of cross-reacting antibodies as a trigger against GalNac is a well-known process since several bacteria and viruses express these same sequences on their surfaces^18^
Hit 3	Complex interactions lead to the formation of circulating macromolecular immune complexes. These complexes can be established between Gd-IgA1 and the specific antiglycan autoantibodies (Hit 2), but also between Gd-IgA1 and soluble CD89, which is generated after IgA-dependent shedding from the surface of myeloid cells.^20^ These complexes are prone to deposit in the mesangium in which they encounter mesangial cell receptors for IgA: transferrin receptor (CD71), integrins a1b1 and a2b2, transglutaminase 2, and b-1,4-galactosyltransferase^4,20^
Hit 4	The deposition of the circulating immune complexes in the glomerular mesangium triggers mesangial cell activation and proliferation, with overproduction of extracellular matrix, cytokines, and chemokines. This leads to podocyte injury and proteinuria, further fueling the recruitment of immune cells and inflammation and promoting glomerular and tubulointerstitial fibrosis^19^

In addition, activation of the complement system by the immune complexes (Hits 3 and 4), especially the alternative and lectin pathways, plays an extremely important role in further triggering glomerular inflammation and kidney injury.^20^ This process is recognized as a key contributing factor to the pathogenesis of IgAN, as it will be further discussed.

### Gd-IgA1-producing B cells

Extensive research has been conducted to explore the mechanisms behind the synthesis of Gd-IgA1, with a particular focus on understanding the involvement of the mucosal immune system and the dysregulated activation of mucosal IgA-committed B cells. Recent evidence has been gathering interest toward B-cell survival factors, specifically a proliferation-inducing ligand (APRIL) and B-cell activating factor (BAFF).^21^ These molecules are cytokines from the tumor necrosis factor (TNF) superfamily and generated by dendritic cells, myeloid cells, and mucosal epithelial cells exposed to antigens. Both cytokines function through receptors known as transmembrane activator and calcium modulator and cyclophilin ligand (CAML) interactor (TACI) and B-cell maturation antigen (BCMA). In addition, BAFF also binds to the BAFF receptor (BAFF-R).^21,22^ Activation of these receptors is crucial for various B-cell-related processes, including survival, maturation, proliferation, and immunoglobulin class switching. At mucosal surfaces, APRIL, acting through TACI, facilitates the class switching of naïve B cells to IgA-producing B cells and is, therefore, a key mediator of IgA and Gd-IgA1.^21,22^ Interestingly, increased APRIL expression in patients with IgAN correlates with high Gd-IgA1 levels and associates with more severe disease presentation and worse outcomes.^21^ Until now, observations have provided evidence that these cytokines contribute to the production of Gd-IgA1 in IgAN and point them as interesting therapeutic targets. However, further investigation is required to clarify the relative contribution of APRIL and BAFF in the pathogenesis of the disease and whether they have distinct roles depending on the stage of progression or subgroup of patients.^21^

### Role of the complement system

The complement system is a key modulator of the adaptive immune response, playing a crucial role in immunosurveillance and maintenance of tissue homeostasis. However, uncontrolled or prolonged activation of the complement cascade significantly contributes to kidney inflammation and glomerular injury.^23^ The formation of immune complexes between Gd-IgA1 and specific immunoglobulins and subsequent tissue deposition are known to trigger local complement activation, specifically the alternative and lectin pathways.^24^ Kidney biopsy samples from patients with IgAN exhibit mesangial deposits of C3, often in conjunction with properdin, which indicates activation of the alternative complement pathway.^20,25^ In a significant proportion of patients, mesangial deposition of C4, particularly C4d, and mannan-binding lectin also suggest the activation of the lectin pathway.^20,24,26^ Overall, glomerular deposits of complement system components have been shown to correlate with worse IgAN prognosis, namely lower estimated glomerular filtration rate (eGFR), higher proteinuria, and more severe histologic damage.^24,27-30^

### Role of the endothelin pathway

Endothelin-1 (ET-1) is an endothelial-derived vasoconstrictor peptide. Nowadays, it is known that ET-1 is produced by practically all cell types in the kidney and acts in an autocrine and/or paracrine manner through the receptors ET_A_R and ET_B_R.^31^ Interestingly, transcriptional and immunostaining approaches performed in kidney biopsies have demonstrated that the endothelin pathway, namely ET-1 and ET_A_R, is activated in patients with IgAN and, moreover, is associated with worse clinical outcomes, such as increased proteinuria and decreased eGFR.^31^ These observations can be explained by the fact that ET_A_R activation induces a plethora of pathophysiological effects in the renal vasculature, glomerulus, and renal tubule. These include platelet aggregation and adhesion, immune cell migration and cytokine production, mesangial and vascular smooth muscle cell contraction, podocyte effacement, cell proliferation, extracellular matrix accumulation and fibrosis, induction of angiotensin II (AngII) and aldosterone, and others.^31^ In experimental models of IgAN, blockade of ET_A_R has been shown to exhibit renal protective effects and, more interestingly, reduce proteinuria in patients with IgAN.^4,31^ The endothelin pathway is, therefore, a major player in IgAN pathogenesis and, currently, one of the most attractive targets for therapeutic applications.

### Genetic background

The pathogenesis of IgAN is deeply affected by genetic factors, and until now, several GWASs involving cohorts from different geographical areas have revealed risk loci associated with the development of this disease. The identified genes are mainly associated with mucosal immunity (*ITGAM*, *ITGAX*, *DEFA*, *LIF*, *OSM*, *HORMAD2*, *MTMR3*, and *TNFSF13* (this latter encoding APRIL)), both innate and adaptive immunities (*CARD9*, *VAV3*, *HLA-DQA1*, *HLA-DQB1*, and *HLA-DRB1*), and complement activation (*CFH*, *CFHR1*, and *CFHR3*).^32-34^ In another study, *VEGFA* and *PKD1L3* gene variants have also been associated with IgAN susceptibility.^35^ More recently, a GWAS with 17 international cohorts identified 16 new loci including *TNFSF4/TNFSF18*, *REL*, *CD28*, *PF4V1*, *LY86*, *LYN*, *ANXA3*, *TNFSF8/TNFSF15*, *REEP3*, *ZMIZ1*, *OVOL1/RELA*, *ETS1*, *IGH*, *IRF8*, *TNFRSF13B*, and *FCAR*.^12^ A specific GWAS brought to light 2 other significant loci encoding enzymes essential for the O-glycosylation of IgA1: *C1GALT1* and *C1GALT1C1*.^36^

These genetic elements reside at the crossroads of various established pathways, indicating pivotal stages in the development of IgAN (preservation of the intestinal mucosal barrier, stimulation of mucosal IgA production, NF-κB signaling, protection against intracellular pathogens, and initiation of complement activation).^36^ Not surprisingly, a genetic risk score based on these loci is higher in Asian populations, moderate in Europeans, and lower in African and African American populations,^2,37^ consistent with reported IgAN prevalence. Specifically, mucosal immunity-related and complement-related genes were more strongly associated with Chinese than with Europeans.^38^

## Histological classification

Histological classification systems are used to describe and categorize disease severity and features. There have been several attempts to classify the histological changes in IgAN to provide clinically relevant information. In the 1980s, Lee et al made the first attempt to predict the progression of renal disease in IgAN through the use of morphological markers.^39^ The study combined the severity of mesangial hypercellularity, glomerular sclerosis, crescents, and tubulointerstitial changes into 5 grades and correlated patients with diffuse proliferative lesions (grade IV) or chronic advanced lesions (grade V) with ESKD. Patients with normal histology or mild-to-moderate lesions (grades II and III) had a benign course without deterioration of renal function.^39^ Nevertheless, this classification was based in a small cohort of 13 patients followed for about 3 years, and subsequent studies led to the refinement of this system. Specifically, a study by Haas and colleagues more than 10 years later focused on a category of patients who did not fit into Lee's classification and who had focal segmental glomerulosclerosis (FSGS)-like lesions without crescents or interstitial chronic damage.^40^ Soon after, Haas established a new histologic grading schema to subclassify patients with IgAN, incorporating features from Lee's classification and of the World Health Organization classification system for lupus nephritis, but specifically recognizing FSGS-like lesions as part of the spectrum of IgAN. It also consisted of 5 classes that were, briefly, organized as follows: minimal histologic lesions (grade I), focal segmental glomerulosclerosis (grade II), proliferative glomerulonephritis in 50% of glomeruli and presence of possible crescents (grade III), proliferative glomerulonephritis in >50% of glomeruli and presence of possible crescents (grade IV), and >40% glomerular sclerosis and/or tubular atrophy (grade V).^40^

Despite these attempts to establish a reliable tool to be used for clinicopathological correlations in IgAN, the studies at the time lacked consistent inclusion criteria and end points and did not provide precise histopathological definitions. This prompted an international group of pathologists and nephrologists to develop a reproducible and clinically relevant IgAN classification system: the Oxford classification.^41^ This was based on a study with clinical data and renal biopsy material from 256 patients with IgAN from 8 countries and 4 continents, finding 4 reproducible histologic variables, independently associated with the clinical outcome.^41^

A large European cohort validation study investigating the utility of this classification system of IgAN (VALIGA) confirmed that M, S, and T lesions independently predicted the loss of eGFR and a lower renal survival.^42^ In patients with eGFR less than 30 ml/min/1.73 m^2^, the M and T lesions independently predicted a poor survival. In those with proteinuria under 0.5 g/day, both M and E lesions were associated with a rise in proteinuria to 1 or 2 g/day or more.^42^ A study involving 500 patients with IgAN showed that the predictive power of the Haas and Oxford classifications concerning renal outcomes were comparable^43^ but alerted for the fact that the relationship between pathological features and responsiveness to immunosuppression was limited because of the retrospective nature of the study.

A revision of the Oxford classification in 2016 led to the inclusion of crescent scores (C) as the fifth variable (MEST-C) (Box 2): C0 (absence of crescents), C1 (crescents in 1%–24% of glomeruli), and C2 (crescents in >25% of glomeruli).^44^ This was due to the observation that patients whose biopsy specimens showed crescents in more than 25% of glomeruli had a worse outcome irrespective of treatment.^44^ The prognostic value of the MEST-C criteria has since been the subject of validation studies, and one, in particular, concluded that there were significant differences in the scoring between local pathologists and a central reviewer, not showing uniform agreement regarding to which areas correlate with independent prognostic value.^45^ Overall, the Oxford MEST-C classification system provides valuable prognostic information at the time of diagnosis but its utility in selecting the management approach for IgAN remains unclear.

Box 2. **MEST-C Score Oxford classification of IgAN.^46^**

**Table:** 

Histological finding	Score
Mesangial hypercellularity (M)	M0: ≤50% of glomeruli showing mesangial hypercellularity
	M1: >50% of glomeruli showing mesangial hypercellularity
Endocapillary hypercellularity (E)	E0: absent
	E1: present in any glomerulus
Segmental glomerulosclerosis (S)	S0: absent
	S1: present in any glomerulus
Tubular atrophy/interstitial fibrosis (T)	T0: 0–25% of the cortical area
	T1: 26–50% of the cortical area
	T2: >50% of the cortical area
Crescents (C)	C0: absent
	C1: 1–24% of glomeruli
	C2: ≥25% of glomeruli

## Diagnosis

The diagnosis of IgAN requires a kidney biopsy demonstrating the presence of dominant or co-dominant mesangial IgA deposits, generally by immunofluorescence or immunoperoxidase staining.^4^ In concomitance with these deposits, C3 and, more infrequently, IgG and IgM can also be found co-deposited in the mesangium and the capillary loops.^4^ Specific IgA1 O-glycoforms and IgA immune complexes may be elevated in the serum, but their use as diagnostic tests is not yet established.^47^

## Modifiable risk factors of disease progression

### Blood pressure

Hypertension is one of the most important factors of accelerated progression of IgAN. Given that hypertension is manageable, there is an obvious need to improve blood pressure in the clinical management of the disease.

The Kidney Disease: Improving Global Outcomes (KDIGO) guidelines from 2021 suggest a systolic blood pressure target of <120 mmHg for adults with high blood pressure and CKD because of the cardiovascular and survival benefits rather than renal benefits.^46^ Given that IgAN is a main cause of CKD, several studies have then addressed the effect of lowering blood pressure during IgAN progression. In a study, Yu et al^48^ showed that systolic blood pressure was independently associated with composite kidney failure events in patients with IgAN, and that lowering it to <140–120 mmHg was renoprotective. Approaches to achieve lower blood pressure targets, such as the use of agents that block the renin-angiotensin-aldosterone (RAAS) system, are critical to properly manage IgAN and arrest its progression to ESKD.

### Proteinuria

Sustained proteinuria is a strong predictor of the rate of renal disease progression and the development of renal failure in IgAN,^16^ and in this context, it has been considered the most promising surrogate end point in IgAN.^49^ A study with 542 patients with IgAN from Canada showed that the rate of eGFR decline was increased with the amount of proteinuria and the 121 patients who had sustained levels of 3 g/day lost renal function 25-fold faster than those with 1 g/day.^50^ This study also stressed the importance of remission or partial remission since the patients whose proteinuria levels decreased from 3 to 1 g/day later developed a similar course to patients who had 1 g/day throughout and managed far better than patients who never achieved remission.^50^ In this work, proteinuria exposure over time (as measured by time-average proteinuria or the average mean of every 6-month period measurements) was the strongest predictor of the rate of renal function decline.^50^ A recent retrospective study engaging a multiethnic cohort of adult patients with IgA nephropathy assessed the effect of the magnitude but also the duration of proteinuria remission in the progression of the disease.^49^ The authors defined proteinuria remission as a ≥25% reduction in proteinuria from the peak value after biopsy and an absolute reduction in proteinuria to <1 g/day. The results showed that among 1864 patients who underwent proteinuria remission, each 3-month period in remission was associated with an additional 9% reduction in the risk of ESKD or a 50% decline in eGFR over a median follow-up of 3.9 years.

Despite the growing body of knowledge regarding proteinuria as a modifiable risk and prognosis factor in IgAN, the fact is that information about disease progression in patients with traditionally considered benign clinical presentations is extremely scarce. This is largely explained by the fact that renal biopsies are generally not performed in this type of patient. Interestingly, a study involving a Caucasian cohort of patients with IgAN with normal renal function and minimal or negative proteinuria (<0.5 g/day) showed no progression of the disease for a median follow-up of 108 months.^13^

However, other investigations into IgAN have indicated that even lower levels of proteinuria can negatively influence the prognosis.^50^ This fact was confirmed recently with the study by Pitcher et al. This study gathered an IgAN cohort of 2299 adults and 140 children from UK National Registry of Rare Kidney Diseases and showed that even when proteinuria is <1 g/day, IgAN cannot be considered a benign condition.^16^ In fact, 30% of patients with a urinary protein-creatinine ratio (UPCR) of 0.44–0.88 g/g, who were traditionally considered at low risk, still had high rates of kidney failure in 10 years, independent of the age of diagnosis.^16^ Importantly, 20% of patients with UPCR <0.44 g/g were also shown to develop kidney failure within 10 years.^16^

Still, presently according to the KDIGO guidelines, a reduction of proteinuria to <1 g/day is considered as a reasonable treatment target in patients with IgA nephropathy.^51^ A certainty in the field is that reducing proteinuria is essential to improve prognosis in these patients. However, the optimal target for proteinuria reduction to attenuate progression of kidney disease is still under debate.

Another interesting aspect is that proteinuria can recur during the long-term course of IgAN. In a retrospective analysis of clinical records of patients with IgAN aged younger than 20 years, approximately 30% of those who had achieved proteinuria remission eventually had recurrence in the following 8 years, regardless of treatment.^52^ Significant factors associated with this recurrence were older onset age and the presence of hematuria after proteinuria remission.^52^ Overall, these data emphasize the need to maintain proteinuria remission to achieve a positive prognostic factor for kidney outcome.

### Hematuria

As previously mentioned, hematuria, either macroscopic or microscopic, is a typical clinical feature of IgAN and is generally present at the time of diagnosis. Persistent microscopic hematuria, measured as time-average hematuria, has recently emerged as a biomarker of disease activity, either in the absence or most notably in the presence of proteinuria.^53^ It is also related to a higher loss of kidney function and progression to ESKD, independent of proteinuria and baseline kidney function.^53^ Moreover, hematuria remission had a significantly beneficial effect in the disease outcome.^54^ However, the value of this parameter is still a matter of debate and not fully supported by international recommendations.^51^ Microscopic hematuria has been frequently disregarded in clinical trials, largely because of technical difficulties. In fact, automated and sensitive systems are required to detect and quantify not only intact but also lysed red blood cells, and currently, flow cytometry particle analyzer systems have shown promise in replacing the laborious and operator-dependent manual microscopy.^53^ In conclusion, the current understanding is that hematuria should be included for enrollment in clinical trials as a readout for drug evaluation, particularly those targeting the immune and inflammatory pathways in IgAN.

## Prognosis

Biomarkers that could predict IgAN course would be of great benefit, but until now, the prognosis of this disease largely depends on the extent of proteinuria, eGFR, and blood pressure.^4^ Histopathological evaluation of biopsies using the Oxford MEST-C classification is also useful to assess prognosis, as previously discussed. Fortunately, an international IgAN prediction tool has been developed, first to predict prognosis at the time of biopsy^55^ and later on to be used up to 2 years to evaluate the risk of a 50% decline in kidney function or kidney failure in 80 months (6.7 years).^56^ The model's formula includes parameters such as eGFR, blood pressure, proteinuria, age, information on treatment (RAAS inhibitors and immune suppression), race, and the scores of the MEST-C classification.^55,56^ This prediction tool has been externally validated and has been updated for use in children. It is also available for clinical use online and in a mobile app calculator.^56^ It has been recommended by the 2021 KDIGO guidelines to be used in risk stratification for IgAN.^51,57^ This tool provides a significant improvement in assisting the prognosis of the disease, but it has not been developed to guide treatment and caution should be advised in that matter.

## Treatment

### Optimized supportive and nonimmunosuppressive drug therapy

**Figure 2.**
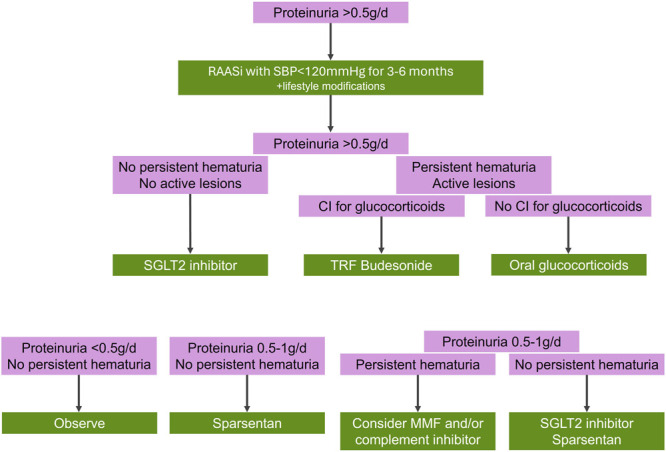
Treatment approaches in patients with IgA nephropathy as proposed by El Karoui et al.^58^ CI, contraindication; MMF, mycophenolate mofetil; TRF, targeted-release formulation; SGLT2, sodium-glucose transporter 2. Adapted from El Karoui K, FC Fervenza, and AS De Vriese. Treatment of IgA nephropathy: A rapidly evolving field. J Am Soc Nephrol. 2024;35(1):103–116,^58^ under the Creative Commons Attribution License 4.0 (CC-BY).

According to the latest KDIGO guidelines, optimized supportive care with lifestyle interventions and nonimmunomodulatory drugs remains the backbone of IgAN management^51^ (Fig. 2). Treatment outcome measures are essentially the reduction of proteinuria and the reduction in the slope of decline in eGFR.^51^

Lifestyle modifications to be implemented include dietary modifications regarding protein and sodium restriction (<2.0 g/day), when appropriate, implementation of a regular physical activity, normalization of body mass, and smoking cessation.^51^ RAAS blockers such as angiotensin-converting enzyme inhibitors (ACEis) or angiotensin II receptor blockers (ARBs) are cornerstones of supportive therapy in patients with IgAN.^51^ These agents reduce intraglomerular pressure and proteinuria, key factors associated with disease progression.^59^ By mitigating glomerular injury and limiting the subsequent inflammatory response and fibrosis, RAAS blockers help preserve renal function and delay the progression of IgAN to ESKD.^59–61^ Clinical studies have demonstrated that RAAS blockade not only lowers proteinuria but also provides a renoprotective effect, underscoring their importance in the therapeutic regimen for patients with IgAN (Table 1).^60–63^

**Table 1:** Overview of landmark clinical trials in the treatment of IgA nephropathy.

	Praga et al^62^	Stop-IgAN^64^	TESTING^65^	NefIgArd^66^	DAPA-CKD^67^	EMPA-KIDNEY^68^	PROTECT^69^
Mechanism of action	ACE inhibitors	Steroid (systemic)	Steroid (systemic)	Steroid (targeted release)	SGLT2 inhibitors	SGLT2 inhibitors	Dual ET_A_R and AT_1_ receptor blocker
Study design	Randomized, prospective	Randomized, open-label, nonblinded	Randomized, placebo controlled, double-blind	Randomized, placebo-controlled, double-blind, phase III	Randomized, placebo controlled, double-blind	Randomized, parallel-group, placebo-controlled, double-blind	Randomized, double-blind, active-controlled, phase III
Regimen	Mean enalapril dose throughout the follow-up was 21 ± 9 mg/d, ranging from 5 to 40 mg/d. In 6 patients (26%) of the treatment group, addition of other antihypertensives was required to achieve the targeted BP (nifedipine in 2, amlodipine in 2, atenolol in 1, diuretics in 2). In the control group, 14 patients (66%) received antihypertensives different from ACE inhibitors during the follow-up, to maintain BP within targeted values (nifedipine in 4, amlodipine in 2, atenolol in 3, amlodipine plus diuretics in 3, and doxazosin in 2)	Control group on supportive care or immunosuppression (level of eGFR >60 ml/min/1.73 m^2^ received a corticosteroid regimen with prednisone and <60 ml/min/1.73 m^2^ received a regimen of prednisolone with cyclophosphamide followed by azathioprine)	Oral methylprednisolone (0.6–0.8 mg/kg/day; maximum 48 mg/day) or matching placebo for 2 months with subsequent weaning over 4–6 months	16-mg/day oral capsules of targeted-release formulation of budesonide or matching placebo for 9 months, followed by a 15-month observational follow-up period off study drug	Dapagliflozin 10 mg or placebo as adjunct to standard care	Empagliflozin (10 mg/day) or matching placebo	Daily oral doses of 400 mg sparsentan vs 300 mg irbesartan (active control)
Sample size	44	162	503	199	270	817	404
Median age (y)	28.9	44.3	36.1	43.5	51.2	53.5	46
Median proteinuria (g/d)	1.85	1.7	1.96	2.3	N/A	N/A	1.8
Median urinary albumin-to-creatinine ratio (mg/g)	N/A	1000	N/A	980	900	577	N/A
Median eGFR (ml/min/1.73m^2^)	N/A	59.3	57.6	55.2	43.8	42.4	57
RAAS blockers during follow-up (%)	In the treatment group	98	N/A	100	N/A	Yes, when applicable	N/A
Previous treatment	N/A	Supportive care for 6 months	At least 3 months of blood pressure control with ACEi/ARB therapy	Maximum tolerated dose of ACEi/ARB therapy for at least 3 months before randomization	ACEi/ARB therapy at least for 4 weeks	92% had ACEi/ARB therapy, and 8.3% had immunosuppression therapy	63.5% had ACEi/ARB therapy at least for 12 weeks
Follow-up	8 y	3 y	4.2 y	2 y	2 y	2 y	2 y
Primary outcome	50% increase in baseline SCr concentrations	Full clinical remission defined as proteinuria with a UPCR of <0.2 and stable renal function with a decrease in the eGFR of <5 ml/min/1.73 m^2^ from the baseline eGFR at the end of the 3-year trial phase	Primary renal outcome: 40% decrease in eGFR, the development of ESKD, or death due to kidney disease	Time-weighted average of eGFR over 2 years	Primary renal outcome: 50% decrease in eGFR or more, the development of ESKD, or death from a kidney disease or cardiovascular cause	Primary renal outcome: 40% decrease in eGFR or more, the development of ESKD, or death from a kidney disease or cardiovascular cause	Primary renal outcome: 40% decrease in eGFR, the development of ESKD, or death due to kidney disease
Risk of experiencing the primary outcome (%)	13% versus 57% in the control group (*P*<.05)	17% versus 5% in the control group (*P*=.01)	7.3% versus 12.1% in the placebo group (*P*<.001)	N/A	4% versus 15% in the placebo group (hazard ratio, 0.29; 95% confidence interval, 0.12, 0.73)	13.7% versus 17.4% in the placebo group (hazard ratio, 0.77; 95% confidence interval, 0.60, 0.98)	8.9% versus 12.9 in the control group (relative risk, 0.68; 95% confidence interval 0.37, 1.24)
Proteinuria/eGFR	Proteinuria decreased significantly by the first year of treatment in the enalapril group, from 2 ± 1.3 g/d to 1.2 ± 1.1 g/d (0–4.5) (−36 ± 40.1%, ranging from −100% to 50% of the baseline values) (*P*<.001) No significant changes were observed in the control group: 1.8 ± 1.5 g/d (0.2–6) (+23 ± 79%; −60 to +216%) by the first year of follow-up (between-group comparison, *P*<.001)	No Absolute eGFR change at 36 months was −4.2 ml/min/1.73 m^2^ versus −4.7 ml/min/1.73 m^2^ in the control group (*P*=.32)	Mean time-average proteinuria of 1.70 g/d versus 2.39 g/d in the placebo group (*P*<.001) Mean eGFR decline was −2.50 ml/min/1.73m^2^/year versus −4.97 ml/min/1.73m^2^/year in the placebo group (*P*=.002)	UACR: At 9 months: 31% reduction versus placebo (*P*=.0005) At 12 months: 54% reduction versus placebo (*P*<.0001) eGFR: At 9 months: 3.87 ml/min/1.73 m^2^ treatment benefit (*P*=.0014) At 12 months: 3.56 ml/min/1.73 m^2^ treatment benefit (*P*=.0106)	UACR: 26% reduction versus placebo (*P*<.001) eGFR: Mean rates of decline −3.5 ml/min/1.73m^2^/year versus −4.7 ml/min/1.73m^2^/year in the placebo group	UACR: 15% lower in the empagliflozin group than in the placebo group (all patients with glomerular disease) (*P*=.05) eGFR: Mean rates of decline were −2.87 ml/min/1.73m^2^/year versus −4.01 ml/min/1.73m^2^/year in the placebo group. (mean changes from 2 months after the first dose of empagliflozin or placebo to the final follow-up visit)	Proteinuria was 49.8% lower in the sparsentan group than in the irbesartan group. (*P*<.0001) eGFR chronic 2-year slope was −2.7 ml/min/1.73 m^2^ per year in the sparsentan group versus −3.8 ml/min/1.73m^2^ per year in the irbesartan group
Adverse effects	Serum potassium levels showed a mild increase at the onset of enalapril treatment, but they were <5.5 mEq/L during the follow-up in all the cases	Numerically higher number of infections, malignant neoplasms, impaired glucose metabolism, and body weight gain in the immunosuppression group	Serious adverse events were reported in 10.9% of participants in the methylprednisolone group compared with 2.8% in the placebo group (hospitalizations and serious infections)	Most reported treatment-emergent adverse events during treatment were peripheral edema (17% patients versus placebo, 4% patients), hypertension (12% versus 3%), muscle spasms (12% versus 4%), acne (11% versus 1%), and headache (10% versus 8%)	Adverse events leading to discontinuation of the study drug were similar in the dapagliflozin (4.4%) and placebo (5.3%) groups. There were fewer serious adverse events with dapagliflozin (16.1%) versus placebo (25.6%)	Ketoacidosis occurred in 6 patients in the empagliflozin group and 1 in the placebo group. There were 28 lower limb amputation events in the empagliflozin group and 19 in the placebo group. These 2 safety outcomes mainly occurred in participants with diabetic kidney disease	Treatment-emergent adverse events were well balanced between sparsentan and irbesartan, with no new safety signals
Comments	After 7 years, the probability of renal survival, estimated based on an increase in SCr to more than 50% above baseline values, was significantly better in the treatment group (92%) than in the control group (55%) (*P*<.05)	Excluded patients with eGFR decline >30% before entry	Stopped prematurely because of severe adverse events	Time-weighted average of eGFR over 2 years showed a statistically significant (*P*<.0001) treatment benefit with budesonide versus placebo (difference 5.05 ml/min/1.73 m^2^)	—	The rate of hospitalization from any cause was lower in the empagliflozin group than in the placebo group (hazard ratio. 0.86; 95% CI 0.78 to 0.95; *P*=.003)	—

ACEi/ARB, angiotensin-converting enzyme inhibitor/angiotensin receptor blocker; BP, blood pressure; eGFR, estimated glomerular filtration rate; ESKD, end-stage kidney disease; N/A, not applicable; RAAS, renin-angiotensin-aldosterone system; sCR, serum creatinine; UACR, urine albumin-creatinine ratio.

Consequently, current guidelines dictate that ACEi or ARB should be used at maximally tolerated dose as first-line in treating patients with IgAN when proteinuria is >0.5 g/day, irrespective of hypertension.^51,59^

New therapies with potential to augment the supportive care approach are emerging, namely the sodium-glucose co-transporter-2 inhibitors (SGLT2is).^70,71^ In 2021, the DAPA-CKD trial investigated the effects of dapagliflozin, a SGLT2i, on the progression of CKD and other major adverse kidney and cardiovascular events in patients with IgAN (Table 1).^67,72^ Based on the results of this trial, dapagliflozin declines albuminuria by 26% and significantly reduced the risk of major adverse kidney events by 71% in patients with IgAN compared with the placebo group.^67^ However, DAPA-CKD enrolled patients who only received ACEi/ARB for 4 weeks, which is much shorter than the treatment course of 3 months recommended by the KDIGO guidelines.^51^ In addition, it was not considered whether the treatment with ACEi/ARB was performed considering the maximal well-tolerated dose, and thus, it might have led to overestimation of the dapagliflozin effect, as maximization of RAAS inhibition also decreases intraglomerular pressure and renoprotective effect.^71^ In addition, the study was not specifically designed to test the hypothesis in patients with IgAN. Patients were only followed for a median of 2.4 years,^67,72^ and long-term follow up studies are still necessary to prove the benefit of these type of drugs in the optimized supportive care of IgAN.^71^ Moreover, attention should be given to the fact that the primary composite end point (composite if a sustained decline in the eGFR of at least 50%, ESKD, or death due to renal or cardiovascular causes) occurred at an unexpected high frequency in the placebo control group.^73^ More recently, the EMPA-KIDNEY trial showed that empagliflozin, another SGLT2i, reduced the risk of kidney disease progression or death due to cardiovascular causes in a broad range of patients with CKD at risk of progression (Table 1).^74^

The endothelin pathway, as previously mentioned, has also emerged as a promising therapeutic target and the focus of new research in the area. Sparsentan is a novel, nonimmunosuppressive, dual ET_A_R and angiotensin II type 1 receptor antagonist, which has recently received US Food and Drug Administration (FDA) accelerated approval owing to its efficacy in reducing proteinuria in the PROTECT trial.^4,69^ In this study, 404 patients with IgAN with persistent proteinuria despite treatment with ACEis or ARBs were randomized 1:1 to receive daily oral doses of sparsentan or the active control irbesartan (ARB) (Table 1).^75^ Specifically, interim results of ongoing phase 3 of this study demonstrated 49.8% reduction of proteinuria from baseline after 36 weeks of treatment compared with the active control arm with irbesartan.^69^ Treatment-emergent adverse events with sparsentan were similar to irbesartan.^69^ The effects were maintained throughout 110 weeks, when proteinuria was 40% lower in the sparsentan group than in the irbesartan group.^76^ The composite kidney failure end point (confirmed 40% eGFR reduction, ESKD, or all-cause mortality) was reached by 9% of patients in the sparsentan group versus 13% of patients in the irbesartan group.^76^ Currently, sparsentan is indicated to reduce proteinuria in adults with primary immunoglobulin IgAN at risk of rapid disease progression, generally a UPCR ≥1.5 g/g. It has not been established whether sparsentan slows kidney function decline in patients with IgAN, and continued approval for this indication may be contingent on verification and description of clinical benefit in a confirmatory clinical trial.

Tonsillectomy has been contemplated as a treatment of IgAN, with the rational of removing a source of pathogens, reducing mucosa-associated lymphoid tissue, and decreasing polymeric IgA synthesis. Nevertheless, its beneficial effect is not consensual and remains controversial in both Asian and Caucasian ethnicities.^77^ In the VALIGA cohort of European patients with IgAN, there was no significant correlation between tonsillectomy and renal function decline, namely in proteinuria, renal end point of 50% reduction in eGFR, and/or ESKD.^78^ A meta-analysis with mostly retrospective studies has suggested that tonsillectomy could be helpful in inducing clinical remission and inhibiting development of ESKD in patients with IgAN.^77^ However, this analysis lacked Caucasian study samples and could not provide any conclusions regarding potential differences between ethnicities.^79^ Thus, more evidence is needed to consider the addition of tonsillectomy to standard clinical treatment of IgAN.

### Immunosuppressive therapy

Current guidelines for the management of IgAN recommend that patients with high risk of progressive loss of kidney function should be considered for immunosuppressive therapy with corticosteroids^51^ (Fig. 2). Systemic glucocorticoids exert anti-inflammatory and immunosuppressive actions and can further improve kidney outcomes. However, recent randomized controlled trials have raised concerns regarding serious infectious and metabolic toxicity.^70,80^ A systematic review gathering 9 randomized controlled trials of corticosteroid therapy for IgAN concluded that a relatively short course of immunosuppression with steroids in IgA nephropathy may reduce the risk of kidney failure by two-thirds compared with supportive therapy or ACEi alone.^81^ Unfortunately, the study also concluded that steroid therapy was associated with 55% higher risk of adverse events.^81^ This systematic review, however, had several limitations regarding the small and short-term studies included and the lack of consistency of data regarding adverse effects.^81^ The randomized controlled trial STOP-IgAN contributed to the controversy regarding the safety and efficacy of this treatment option (Table 1). This trial found no difference in the annual decline of eGFR between the supportive care group and the immunosuppression group over a median follow-up of 7.4 years.^82,83^ Although the addition of immunosuppressive therapy to supportive care was superior to supportive care alone in inducing remission of proteinuria in a proportion of patients, it did not translate into long-term beneficial effects on kidney outcomes, as there was no significant difference in the annual decline in eGFR between the 2 groups. Moreover, the corticosteroid group experienced a higher incidence of adverse events, including severe infections (including 1 death due to sepsis), impaired glucose tolerance, and weight gain.^82^ This study excluded patients with proteinuria >3.5 g/day and those who had a very rapid decrease in the eGFR during the run-in phase and, thus, cannot comment on the effectiveness of the immunosuppressive treatment in subgroups that could likely benefit from it. In addition, histological findings were not considered throughout the trial.

Meanwhile, the TESTING trial was a multicenter, randomized clinical trial that gathered 750 patients with IgAN with proteinuria >1 g/day and eGFR of 20–120 ml/min/1.73 m^2^ after at least 3 months of blood pressure control with RAAS blockade (Table 1).^84^ In this trial, patients were randomized 1:1 to oral corticosteroid (methylprednisolone) or placebo groups for 2 months, but after 2.1 years of median follow-up, the study was discontinued because of excess serious adverse events in the treatment groups, namely severe infections and 2 deaths.^84^ Nevertheless, until trial cessation, the results were consistent with a potential renal benefit, since the primary renal outcome (ESKD, death due to kidney failure, or a 40% decrease in eGFR) occurred in 5.9% in the methylprednisolone group vs 15.9% in the placebo group.^84^ A recent follow-up study of the TESTING trial investigated the effect of reduced-dose oral corticosteroid therapy with concomitant antibiotic prophylaxis on both the eGFR and proteinuria after a mean follow-up of 4.2 years.^65^ Results supported the renoprotective effects of the steroid treatment and the improved safety profile, but 1 infection-related death still occurred in the treatment group.^65^

Both the STOP-IgAN and the TESTING trials recognized the increased risk of serious infectious adverse effects associated with corticosteroid therapy. Nonetheless, while proteinuria was successfully decreased in both studies, the effect was transient in the STOP-IgAN trial. Interestingly, different outcomes in these studies could have been related to the fact that the STOP-IgAN trial only included Caucasian patients,^82^ whereas 95% of participants in the TESTING trial were of Asian ancestry, which is a population known to have more aggressive disease, higher baseline proteinuria levels, and a more rapid decline in kidney function.^84^

Current KDIGO guidelines regarding IgAN advocate that if after at least 90 days of optimized supportive care (including RAAS blockade), proteinuria stays >0.75 g/day and eGFR is >30 ml/min/1.73m^2^, patients should be considered for a 6-month course of steroid therapy (Grade 2B) or the enrollment in a therapeutic clinical trial.^51^ However, the guidelines stress that the clinical benefit of steroids in IgAN is still not established and that it should be avoided in patients with eGFR <30 ml/min/1.73 m^2^, diabetes, obesity, secondary disease, active peptic ulceration, uncontrolled psychiatric disease, or severe osteoporosis.^51^

Apart from glucocorticoids, other immunosuppressive therapies, such as azathioprine, cyclophosphamide, calcineurin inhibitors, and rituximab, are not currently recommended by the 2021 KDIGO guidelines,^51^ but other agents are currently under investigation.

Mycophenolate mofetil (MMF) is a potent immunosuppressive agent that is relatively selective for lymphocytes and strongly inhibits antibody production by B cells.^85^ It can be considered as an alternative to steroids or as a steroid-sparing agent in Chinese patients, since adverse events associated with MMF are lower than those observed with systemic glucocorticoids.^85–88^ Until now, only Chinese studies have showed that, in patients with IgAN with mild histologic lesions and persistent proteinuria despite maximal angiotensin blockade, MMF treatment may result in transient and partial remission of proteinuria in the short term and renoprotection in the long term (6 years of follow-up).^88,89^ A recent open-label 3-year trial with 170 Chinese patients with IgAN and persistent proteinuria were randomized to MMF therapy plus supportive care.^85^ Among patients with IgAN who were at high risk of progression, the trial found that the addition of MMF to standard care significantly reduced the risk of doubling of serum creatinine, ESKD, or death due to kidney or cardiovascular causes compared with standard care alone.^85^ It was concluded that addition of MMF to optimized standard care could be an alternative therapy for patients at risk of progressive disease and those not appropriate for steroid therapy.^85^ This trial excluded patients with proteinuria >3.5 g/day and, therefore, did not evaluate the efficacy of the treatment in this population.^85^ Contrary to these studies in the Chinese population, Belgian and North American trials that recruited patients with more advanced disease did not report any benefit from adding MMF to standard care.^86,87,90^ Owing to the lack of evidence regarding the effectiveness of MMF in the context of progressive IgAN, the 2021 KDIGO guidelines do not recommended its use in non-Chinese patients but suggests that it may be used as a glucocorticoid-sparing agent in Chinese patients.^51^

Hydroxychloroquine, a TLR9, TLR8, and TLR7 inhibitor, has been evaluated for the treatment of IgAN in a single-center Chinese study with patients with proteinuria of 0.75–3.5 g/day and eGFR >30 ml/min/1.73 m^2^ who were receiving optimized RAAS inhibitor therapy.^91^ Hydroxychloroquine in addition to optimized standard care effectively reduced proteinuria over 6 months without evidence of adverse events.^91^ Long-term data indicate that hydroxychloroquine with conventional RAAS inhibitor therapy can reduce proteinuria by 50% within 24 months, without significant decline in eGFR and major adverse events, underlining hydroxychloroquine as a potentially effective supportive treatment for Chinese patients in the long term.^92^

The intestinal mucosa immune system has been proposed as the main site of IgA production in the body, and a new targeted release formulation of the oral glucocorticoid budesonide (TRF-budesonide), which is preferentially released in the ileal gut-associated lymphoid system, has been shown to reduce the activity of IgAN,^93,94^ without the adverse effects related to systemic steroid therapy. The therapeutic potential of TRF-budesonide was assessed in an international, multicenter, double-blind phase III trial designated NefIgArd (Table 1).^93^ Patients with IgAN enrolled in the study had persistent proteinuria (>1 g/day), despite optimized supportive care, and an eGFR of 35–90 ml/min/1.73 m^2^ and had received the maximum tolerated dose of an RAAS inhibition therapy for at least 3 months before randomization.^93^ After 9 months, proteinuria was 27% lower in the TRF-budesonide-treated group compared with placebo, along with a benefit in eGFR preservation corresponding to a 3.87 ml/min/1.73 m^2^ difference versus placebo.^93^ Adverse events were mostly mild to moderate in severity and reversible.^93^ Follow-up data of 2 years recently concluded that the treatment with TRF-budesonide demonstrated a clinically relevant reduction in eGFR decline and a durable reduction in proteinuria versus placebo.^94^ In the treatment group, UPCR decrease reached a maximal effect 3 months after treatment discontinuation (−49.7% compared with placebo).^94^ However, after these 3 months until the end of the observational follow-up period (24 months), the percentage reduction in UPCR decreased from 49.7% to 30.1% (Nefecon versus placebo).^94^ TRF-budesonide was granted accelerated approval by the FDA, and it is the first approved treatment of IgAN.^4^

## New therapeutic approaches in the management of IgAN

New milestones for the therapy of IgAN have been set in the past 2 years. We foresee that the guidelines for the management of IgAN will incorporate in the future the use of SGLT2 inhibitors and endothelin receptor antagonists, such as sparsentan, as options for the optimized supportive care approach and the inclusion of TRF-budesonide therapy for patients with high risk of progressive disease.^58,80,95^ It is also important to keep in mind that patients with IgAN with proteinuria levels <1 g/day remain at risk of kidney failure. This should be taken into consideration in future recommendations concerning the threshold for kidney biopsy, treatment options, and even enrollment in clinical trials.^96,97^

The increased recognition of proteinuria reduction as a surrogate marker of ESKD has greatly enhanced the viability of conducting clinical trials in IgAN. Presently, numerous trials are underway, focusing on various novel therapeutic targets. These are focused, essentially, on complement inhibition and interference with B-cell maturation and proliferation.

About targeting B cells, and consequently production of Gd-IgA1 and anti-Gd-IgA1 antibodies, numerous studies are on the way. Although the chimeric monoclonal anti-CD20 antibody rituximab reduced circulating B cells, it did not demonstrate any benefit in IgAN, as it failed to lower serum Gd-IgA1 and anti-Gd-IgA1 levels.^98^ These results led to the hypothesis that CD20^+^ cells might not be the exclusive producers of Gd-IgA1. In fact, a current clinical trial is investigating felzartamab as a targeting agent of CD38^+^ cells (NCT05065970). There are also ongoing strategies involving the inhibition of BAFF and APRIL, through either monoclonal antibodies such as sibeprenlimab (NCT04287985), zigakibart (NCT04287985), and blisibimod (NCT02062684) or inhibitors such as atacicept (NCT04716231), telitacicept (NCT05799287), and fostamatinib (NCT02112838). In fact, a recent randomized, double-blind placebo-controlled, phase 2 trial reported that 12 months of treatment with sibeprenlimab (a humanized IgG2 monoclonal antibody that binds to and neutralizes APRIL) resulted in a significantly greater decrease in proteinuria and lower eGFR decline in patients with IgAN than in placebo.^99^

Considering complement system-directed therapies, there are numerous ongoing phase II/III trials but those with the most promising results concern narsoplimab, an IgG4 monoclonal antibody that inhibits MBL-associated serine protease 2 which is the key effector enzyme of the lectin pathway, and iptacopan, an oral selective inhibitor of factor B of the alternative pathway. Narsoplimab showed substantial reduction of proteinuria in a phase II clinical trial (NCT02682407), but the interim analysis of the phase III trial yielded unfavorable results, leading to the discontinuation of the trial (NCT03608033). In the phase II trial, iptacopan led to continuous decrease in proteinuria and strong inhibition of alternative pathway activity over 6 months,^100^ and a phase III study (APPLAUSE-IgAN) is currently ongoing (NCT04578834). The results of these and other ongoing trials hold the potential to significantly alter the future of the treatment paradigm for IgAN.

## Future perspectives

Imperative tasks for the future are to identify the best treatment combinations and biomarkers to guide therapeutic choices and allow personalized and tailored management of IgAN. While we anticipate the development of future strategies that target the upstream production of Gd-IgA, specific antibodies, and/or the immune complexes, multidrug approaches that target the various pathogenic processes of the disease may be needed in the treatment of IgAN in the foreseeable future. At the moment, the treatment paradigm is guided by levels of proteinuria and eGFR, which are nonspecific and frequent markers of kidney damage. Advances in reliable, noninvasive biomarkers of disease activity and remission will be crucial to shape clinical practice. The availability of such markers, for example, assessing the levels of Gd-IgA1 or IgG/IgA autoantibodies, could help define an appropriate treatment strategy, monitor disease activity during follow-up, and predict the need for retreatment.^19,80^ Patients with IgAN have relapsing episodes, and unfortunately, there are still very limited data on second-line therapy, retreatment for these situations, or need to perform a second renal biopsy.^101^

## Conclusions

New and exciting advances in IgA management are expected in the near future. The growing body of knowledge on the pathophysiology of this heterogeneous and progressive disease is leading to a plethora of clinically relevant groundbreaking approaches. Novel treatments, biomarkers, and other integrative tools will soon be available to prevent disease progression and increase the life expectancy of patients with IgAN.

## References

[1] McGrogan A Franssen CF de Vries CS. The incidence of primary glomerulonephritis worldwide: a systematic review of the literature. Nephrol Dial Transpl. 2011;26:414–30. 10.1093/ndt/gfq665 21068142

[2] Lee M Suzuki H Nihei Y Matsuzaki K Suzuki Y. Ethnicity and IgA nephropathy: worldwide differences in epidemiology, timing of diagnosis, clinical manifestations, management and prognosis. Clin Kidney J. 2023;16(Suppl 2):ii1–8. 38053973 10.1093/ckj/sfad199 PMC10695519

[3] Lai KN Tang SCW Schena FP . IgA nephropathy. Nat Rev Dis Primers. 2016;2:16001. 27189177 10.1038/nrdp.2016.1

[4] Stamellou E Seikrit C Tang SCW . IgA nephropathy. Nat Rev Dis Primers. 2023;9:67. 38036542 10.1038/s41572-023-00476-9

[5] Zhang H Barratt J. Is IgA nephropathy the same disease in different parts of the world? Semin Immunopathol. 2021;43:707–15. 34417628 10.1007/s00281-021-00884-7

[6] Kiryluk K Freedberg DE Radhakrishnan J . Global incidence of IgA nephropathy by race and ethnicity: a systematic review. Kidney360. 2023;4:1112–22. 37227924 10.34067/KID.0000000000000165 PMC10476677

[7] Rodrigues JC Haas M Reich HN. IgA nephropathy. Clin J Am Soc Nephrol. 2017;12:677–86. 28159829 10.2215/CJN.07420716 PMC5383386

[8] Willey CJ Coppo R Schaefer F Mizerska-Wasiak M Mathur M Schultz MJ. The incidence and prevalence of IgA nephropathy in Europe. Nephrol Dial Transpl. 2023;38:2340–9. 10.1093/ndt/gfad082 PMC10539204 37156519

[9] Gabinete de Registo de Biópsias Renais, Registo Nacional de Biópsias Renais. Lisbon, Portugal: Sociedade Portuguesa de Nefrologia; 2022.

[10] Schena FP Nistor I. Epidemiology of IgA nephropathy: a global perspective. Semin Nephrol. 2018;38:435–42. 30177015 10.1016/j.semnephrol.2018.05.013

[11] Okpechi IG Ameh OI Bello AK Ronco P Swanepoel CR Kengne AP. Epidemiology of histologically proven glomerulonephritis in Africa: a systematic review and meta-analysis. PLoS One. 2016;11:e0152203. 27011216 10.1371/journal.pone.0152203 PMC4806979

[12] Kiryluk K Sanchez-Rodriguez E Zhou XJ . Genome-wide association analyses define pathogenic signaling pathways and prioritize drug targets for IgA nephropathy. Nat Genet. 2023;55:1091–105. 37337107 10.1038/s41588-023-01422-x PMC11824687

[13] Gutierrez E Zamora I Ballarín JA . Long-term outcomes of IgA nephropathy presenting with minimal or no proteinuria. J Am Soc Nephrol. 2012;23:1753–60. 22956820 10.1681/ASN.2012010063 PMC3458461

[14] Le W Liang S Chen H . Long-term outcome of IgA nephropathy patients with recurrent macroscopic hematuria. Am J Nephrol. 2014;40:43–50. 24994520 10.1159/000364954

[15] Coppo R. Treatment of IgA nephropathy: recent advances and prospects. Nephrol Ther. 2018;14(Suppl 1):S13–21. 29606258 10.1016/j.nephro.2018.02.010

[16] Pitcher D Braddon F Hendry B . Long-term outcomes in IgA nephropathy. Clin J Am Soc Nephrol. 2023;18:727–38. 37055195 10.2215/CJN.0000000000000135 PMC10278810

[17] Fassbinder W Brunner FP Brynger H . Combined report on regular dialysis and transplantation in Europe, XX, 1989*.* Nephrol Dial Transplant. 1991;6(Suppl 1):5–35. 2038432

[18] Magistroni R D'Agati VD Appel GB Kiryluk K. New developments in the genetics, pathogenesis, and therapy of IgA nephropathy. Kidney Int. 2015;88:974–89. 26376134 10.1038/ki.2015.252 PMC4653078

[19] Knoppova B Reily C Maillard N . The origin and activities of IgA1-containing immune complexes in IgA nephropathy. Front Immunol. 2016;7:117. 27148252 10.3389/fimmu.2016.00117 PMC4828451

[20] Tesar V Radhakrishnan J Charu V Barratt J. Challenges in IgA nephropathy management: an era of complement inhibition. Kidney Int Rep. 2023;8:1730–40. 37705895 10.1016/j.ekir.2023.06.010 PMC10496078

[21] Yeo SC Barratt J. The contribution of a proliferation-inducing ligand (APRIL) and other TNF superfamily members in pathogenesis and progression of IgA nephropathy. Clin Kidney J. 2023;16(Suppl 2):ii9–18. 38053976 10.1093/ckj/sfad200 PMC10695512

[22] Cheung CK Barratt J Carroll K . Targeting APRIL in the treatment of IgA nephropathy. Clin J Am Soc Nephrol. 2024;19:394–8. 37801688 10.2215/CJN.0000000000000338 PMC10937009

[23] Duval A Caillard S Frémeaux-Bacchi V. The complement system in IgAN: mechanistic context for therapeutic opportunities. Nephrol Dial Transpl. 2023;38:2685–93. 10.1093/ndt/gfad140 37385820

[24] Caravaca-Fontan F Gutiérrez E Sevillano ÁM Praga M. Targeting complement in IgA nephropathy. Clin Kidney J. 2023;16(Suppl 2):ii28–39. 38053977 10.1093/ckj/sfad198 PMC10695513

[25] Maillard N Wyatt RJ Julian BA . Current understanding of the role of complement in IgA nephropathy. J Am Soc Nephrol. 2015;26:1503–12. 25694468 10.1681/ASN.2014101000 PMC4483595

[26] Faria B Henriques C Matos AC Daha MR Pestana M Seelen M. Combined C4d and CD3 immunostaining predicts immunoglobulin (Ig)A nephropathy progression. Clin Exp Immunol. 2015;179:354–61. 25267249 10.1111/cei.12461 PMC4298411

[27] Wu J Hu Z Wang Y . Severe glomerular C3 deposition indicates severe renal lesions and a poor prognosis in patients with immunoglobulin A nephropathy. Histopathology. 2021;78:882–95. 33336446 10.1111/his.14318

[28] Xie M Zhu Y Wang X . Predictive prognostic value of glomerular C3 deposition in IgA nephropathy. J Nephrol. 2023;36:495–505. 35781866 10.1007/s40620-022-01363-4

[29] Jiang Y Zan J Shi S . Glomerular C4d deposition and kidney disease progression in IgA nephropathy: a systematic review and meta-analysis. Kidney Med. 2021;3:1014–21. 34939010 10.1016/j.xkme.2021.06.009 PMC8664744

[30] Neves P Souza RA Torres FM . Evidences of histologic thrombotic microangiopathy and the impact in renal outcomes of patients with IgA nephropathy. PLoS One. 2020;15:e0233199. 33147224 10.1371/journal.pone.0233199 PMC7641451

[31] Kohan DE Barratt J Heerspink HJL . Targeting the endothelin A receptor in IgA nephropathy. Kidney Int Rep. 2023;8:2198–210. 38025243 10.1016/j.ekir.2023.07.023 PMC10658204

[32] Kiryluk K Li Y Scolari F . Discovery of new risk loci for IgA nephropathy implicates genes involved in immunity against intestinal pathogens. Nat Genet. 2014;46:1187–96. 25305756 10.1038/ng.3118 PMC4213311

[33] Zhang YM Zhou XJ Zhang H. What genetics tells us about the pathogenesis of IgA nephropathy: the role of immune factors and infection. Kidney Int Rep. 2017;2:318–31. 29142962 10.1016/j.ekir.2017.02.005 PMC5678660

[34] Gharavi AG Kiryluk K Choi M . Genome-wide association study identifies susceptibility loci for IgA nephropathy. Nat Genet. 2011;43:321–7. 21399633 10.1038/ng.787 PMC3412515

[35] Li M Wang YN Wang L . Genome-wide association analysis of protein-coding variants in IgA nephropathy. J Am Soc Nephrol. 2023;34:1900–13. 37787447 10.1681/ASN.0000000000000222 PMC10631603

[36] Kiryluk K Li Y Moldoveanu Z . GWAS for serum galactose-deficient IgA1 implicates critical genes of the O-glycosylation pathway. PLoS Genet. 2017;13:e1006609. 28187132 10.1371/journal.pgen.1006609 PMC5328405

[37] Kiryluk K Novak J Gharavi AG. Pathogenesis of immunoglobulin A nephropathy: recent insight from genetic studies. Annu Rev Med. 2013;64:339–56. 23072577 10.1146/annurev-med-041811-142014 PMC3921622

[38] Li M Wang L Shi DC . Genome-wide meta-analysis identifies three novel susceptibility loci and reveals ethnic heterogeneity of genetic susceptibility for IgA nephropathy. J Am Soc Nephrol. 2020;31:2949–63. 32912934 10.1681/ASN.2019080799 PMC7790208

[39] Lee SM Rao VM Franklin WA . IgA nephropathy: morphologic predictors of progressive renal disease. Hum Pathol. 1982;13:314–22. 7076216 10.1016/s0046-8177(82)80221-9

[40] Haas M. IgA nephropathy histologically resembling focal-segmental glomerulosclerosis: a clinicopathologic study of 18 cases. Am J Kidney Dis. 1996;28:365–71. 8804234 10.1016/s0272-6386(96)90493-x

[41] Working Group of the International IgA Nephropathy Network and the Renal Pathology Society, Cattran DC Coppo R Cook HT . The Oxford classification of IgA nephropathy: rationale, clinicopathological correlations, and classification. Kidney Int. 2009;76:534–45. 19571791 10.1038/ki.2009.243

[42] Coppo R Troyanov S Bellur S . Validation of the Oxford classification of IgA nephropathy in cohorts with different presentations and treatments. Kidney Int. 2014;86:828–36. 24694989 10.1038/ki.2014.63 PMC4184028

[43] Park KS Han SH Kie JH . Comparison of the Haas and the Oxford classifications for prediction of renal outcome in patients with IgA nephropathy. Hum Pathol. 2014;45:236–43. 24439222 10.1016/j.humpath.2013.08.019

[44] Trimarchi H Barratt J Cattran DC . Oxford classification of IgA nephropathy 2016: an update from the IgA nephropathy classification working group. Kidney Int. 2017;91:1014–21. 28341274 10.1016/j.kint.2017.02.003

[45] Bellur SS Roberts ISD Troyanov S . Reproducibility of the Oxford classification of immunoglobulin A nephropathy, impact of biopsy scoring on treatment allocation and clinical relevance of disagreements: evidence from the VALidation of IGA study cohort. Nephrol Dial Transpl. 2019;34:1681–90. 10.1093/ndt/gfy337 30561721

[46] Cheung AK Chang TI Cushman WC . Executive summary of the KDIGO 2021 clinical practice guideline for the management of blood pressure in chronic kidney disease. Kidney Int. 2021;99:559–69. 33637203 10.1016/j.kint.2020.10.026

[47] Selvaskandan H Shi S Twaij S Cheung CK Barratt J. Monitoring immune responses in IgA nephropathy: biomarkers to guide management. Front Immunol. 2020;11:572754. 33123151 10.3389/fimmu.2020.572754 PMC7572847

[48] Yu G Cheng J Jiang Y Li H Li X Chen J. Intensive systolic blood pressure lowering and kidney disease progression in IgA nephropathy: a cohort study. Front Med (Lausanne). 2022;9:813603. 35252253 10.3389/fmed.2022.813603 PMC8890476

[49] Canney M Barbour SJ Zheng Y . Quantifying duration of proteinuria remission and association with clinical outcome in IgA nephropathy. J Am Soc Nephrol. 2021;32:436–47. 33514642 10.1681/ASN.2020030349 PMC8054888

[50] Reich HN Troyanov S Scholey JW Cattran DC, Toronto Glomerulonephritis Registry. Remission of proteinuria improves prognosis in IgA nephropathy. J Am Soc Nephrol. 2007;18:3177–83. 17978307 10.1681/ASN.2007050526

[51] Rovin BH Adler SG Barratt J . Executive summary of the KDIGO 2021 guideline for the management of glomerular diseases. Kidney Int. 2021;100:753–79. 34556300 10.1016/j.kint.2021.05.015

[52] Shima Y Mukaiyama H Tanaka Y . Factors related to recurrence of proteinuria in childhood IgA nephropathy. Pediatr Nephrol. 2024;39:463–71. 37594578 10.1007/s00467-023-06116-4

[53] Zand L Fervenza FC Coppo R. Microscopic hematuria as a risk factor for IgAN progression: considering this biomarker in selecting and monitoring patients. Clin Kidney J. 2023;16(Suppl 2):ii19–27. 38053974 10.1093/ckj/sfad232 PMC10695511

[54] Sevillano AM Gutiérrez E Yuste C . Remission of hematuria improves renal survival in IgA nephropathy. J Am Soc Nephrol. 2017;28:3089–99. 28592423 10.1681/ASN.2017010108 PMC5619972

[55] Barbour SJ Coppo R Zhang H . Evaluating a new international risk-prediction tool in IgA nephropathy. JAMA Intern Med. 2019;179:942–52. 30980653 10.1001/jamainternmed.2019.0600 PMC6583088

[56] Barbour SJ Coppo R Zhang H . Application of the international IgA nephropathy prediction tool one or two years post-biopsy. Kidney Int. 2022;102:160–72. 35490842 10.1016/j.kint.2022.02.042

[57] Kidney Disease Improving Global Outcomes KDIGO Glomerular Diseases Work Group. KDIGO 2021 clinical practice guideline for the management of glomerular diseases. Kidney Int. 2021;100:S1–276. 34556256 10.1016/j.kint.2021.05.021

[58] El Karoui K Fervenza FC De Vriese AS. Treatment of IgA nephropathy: a rapidly evolving field. J Am Soc Nephrol. 2024;35:103–16. 37772889 10.1681/ASN.0000000000000242 PMC10786616

[59] Bagchi S Mani K Swamy A . Supportive management of IgA nephropathy with renin-angiotensin blockade, the AIIMS primary IgA nephropathy cohort (APPROACH) study. Kidney Int Rep. 2021;6:1661–8. 34169207 10.1016/j.ekir.2021.02.018 PMC8207308

[60] Ji Y Yang K Xiao B . Efficacy and safety of angiotensin-converting enzyme inhibitors/angiotensin receptor blocker therapy for IgA nephropathy: a meta-analysis of randomized controlled trials. J Cel Biochem. 2019;120:3689–95. 10.1002/jcb.27648 30270542

[61] Cheng J Zhang W Zhang XH He Q Tao XJ Chen JH. ACEI/ARB therapy for IgA nephropathy: a meta analysis of randomised controlled trials. Int J Clin Pract. 2009;63:880–8. 19490198 10.1111/j.1742-1241.2009.02038.x

[62] Praga M Gutiérrez E González E Morales E Hernández E. Treatment of IgA nephropathy with ACE inhibitors: a randomized and controlled trial. J Am Soc Nephrol. 2003;14:1578–83. 12761258 10.1097/01.asn.0000068460.37369.dc

[63] Zhao Y Fan H Bao BY. Efficacy and safety of renin-angiotensin aldosterone system inhibitor in patients with IgA nephropathy: a meta-analysis of randomized controlled trials. Iran J Public Health. 2019;48:1577–88. 31700813 PMC6825685

[64] Rauen T Wied S Fitzner C, et al. After ten years of follow-up, no difference between supportive care plus immunosuppression and supportive care alone in IgA nephropathy. Kidney Int. 2020;98:1044–1052. In press. 32450154 10.1016/j.kint.2020.04.046

[65] Lv J Wong MG Hladunewich MA . Effect of oral methylprednisolone on decline in kidney function or kidney failure in patients with IgA nephropathy: the TESTING randomized clinical trial. JAMA. 2022;327:1888–98. 35579642 10.1001/jama.2022.5368 PMC9115617

[66] Lafayette R Kristensen J Stone A, et al. Efficacy and safety of a targeted-release formulation of budesonide in patients with primary IgA nephropathy (NefIgArd): 2-year results from a randomised phase 3 trial. Lancet. 2023;402:859–870. 37591292 10.1016/S0140-6736(23)01554-4

[67] Wheeler DC Toto RD Stefánsson BV . A pre-specified analysis of the DAPA-CKD trial demonstrates the effects of dapagliflozin on major adverse kidney events in patients with IgA nephropathy. Kidney Int. 2021;100:215–24. 33878338 10.1016/j.kint.2021.03.033

[68] EMPA-KIDNEY Collaborative Group. Impact of primary kidney disease on the effects of empagliflozin in patients with chronic kidney disease: secondary analyses of the EMPA-KIDNEY trial. Lancet Diabetes Endocrinol. 2024;12:51–60. 38061372 10.1016/S2213-8587(23)00322-4 PMC7618536

[69] Heerspink HJL Radhakrishnan J Alpers CE . Sparsentan in patients with IgA nephropathy: a prespecified interim analysis from a randomised, double-blind, active-controlled clinical trial. Lancet. 2023;401:1584–94. 37015244 10.1016/S0140-6736(23)00569-X

[70] Gleeson PJ O'Shaughnessy MM Barratt J. IgA nephropathy in adults-treatment standard. Nephrol Dial Transpl. 2023;38:2464–73. 10.1093/ndt/gfad146 PMC10794095 37418237

[71] Maixnerova D Hartinger J Tesar V. Expanding options of supportive care in IgA nephropathy. Clin Kidney J. 2023;16(Suppl 2):ii47–54. 38053975 10.1093/ckj/sfad201 PMC10695500

[72] Yasuda H Isobe S. Dapagliflozin in patients with chronic kidney disease. N Engl J Med. 2021;384:389. 10.1056/NEJMc2032809 33503361

[73] Barratt J Floege J. SGLT-2 inhibition in IgA nephropathy: the new standard of care? Kidney Int. 2021;100:24–6. 33878337 10.1016/j.kint.2021.04.002

[74] The EMPA-KIDNEY Collaborative Group. Empagliflozin in patients with chronic kidney disease. N Engl J Med. 2023;388:117–27. 36331190 10.1056/NEJMoa2204233 PMC7614055

[75] Barratt J Rovin B Wong MG . IgA nephropathy patient baseline characteristics in the sparsentan PROTECT study. Kidney Int Rep. 2023;8:1043–56. 37180506 10.1016/j.ekir.2023.02.1086 PMC10166729

[76] Rovin BH Barratt J Heerspink HJL . Efficacy and safety of sparsentan versus irbesartan in patients with IgA nephropathy (PROTECT): 2-year results from a randomised, active-controlled, phase 3 trial. Lancet. 2023;402:2077–90. 37931634 10.1016/S0140-6736(23)02302-4

[77] Duan J Liu D Duan G Liu Z. Long-term efficacy of tonsillectomy as a treatment in patients with IgA nephropathy: a meta-analysis. Int Urol Nephrol. 2017;49:103–12. 27722990 10.1007/s11255-016-1432-7

[78] Feehally J Coppo R Troyanov S . Tonsillectomy in a European cohort of 1,147 patients with IgA nephropathy. Nephron. 2016;132:15–24. 26586175 10.1159/000441852

[79] Zheng Y Wang Y Liu S . Potential blood pressure goals in IgA nephropathy: prevalence, awareness, and treatment rates in chronic kidney disease among patients with hypertension in China (PATRIOTIC) study. Kidney Blood Press Res. 2018;43:1786–95. 30504700 10.1159/000495636

[80] Locatelli F Del Vecchio L Ponticelli C. Systemic and targeted steroids for the treatment of IgA nephropathy. Clin Kidney J. 2023;16(Suppl 2):ii40–6. 38053978 10.1093/ckj/sfad224 PMC10695509

[81] Lv J Xu D Perkovic V . Corticosteroid therapy in IgA nephropathy. J Am Soc Nephrol. 2012;23:1108–16. 22539830 10.1681/ASN.2011111112 PMC3358763

[82] Rauen T Eitner F Fitzner C . Intensive supportive care plus immunosuppression in IgA nephropathy. N Engl J Med. 2015;373:2225–36. 26630142 10.1056/NEJMoa1415463

[83] Rauen T Wied S Fitzner C . After ten years of follow-up, no difference between supportive care plus immunosuppression and supportive care alone in IgA nephropathy. Kidney Int. 2020;98:1044–52. 32450154 10.1016/j.kint.2020.04.046

[84] Lv J Zhang H Wong MG . Effect of oral methylprednisolone on clinical outcomes in patients with IgA nephropathy: the TESTING randomized clinical trial. JAMA. 2017;318:432–42. 28763548 10.1001/jama.2017.9362 PMC5817603

[85] Hou FF Xie D Wang J . Effectiveness of mycophenolate mofetil among patients with progressive IgA nephropathy: a randomized clinical trial. JAMA Netw Open. 2023;6:e2254054. 36745456 10.1001/jamanetworkopen.2022.54054 PMC12578496

[86] Maes BD Oyen R Claes K . Mycophenolate mofetil in IgA nephropathy: results of a 3-year prospective placebo-controlled randomized study. Kidney Int. 2004;65:1842–9. 15086925 10.1111/j.1523-1755.2004.00588.x

[87] Frisch G Lin J Rosenstock J . Mycophenolate mofetil (MMF) vs placebo in patients with moderately advanced IgA nephropathy: a double-blind randomized controlled trial. Nephrol Dial Transpl. 2005;20:2139–45. 10.1093/ndt/gfh974 16030050

[88] Tang SC Tang AWC Wong SSH Leung JCK Ho YW Lai KN. Long-term study of mycophenolate mofetil treatment in IgA nephropathy. Kidney Int. 2010;77:543–9. 20032964 10.1038/ki.2009.499

[89] Tang S Leung JCK Chan LYY . Mycophenolate mofetil alleviates persistent proteinuria in IgA nephropathy. Kidney Int. 2005;68:802–12. 16014059 10.1111/j.1523-1755.2005.00460.x

[90] Hogg RJ Bay RC Jennette JC . Randomized controlled trial of mycophenolate mofetil in children, adolescents, and adults with IgA nephropathy. Am J Kidney Dis. 2015;66:783–91. 26209543 10.1053/j.ajkd.2015.06.013

[91] Liu LJ Yang YZ Shi SF . Effects of hydroxychloroquine on proteinuria in IgA nephropathy: a randomized controlled trial. Am J Kidney Dis. 2019;74:15–22. 30922594 10.1053/j.ajkd.2019.01.026

[92] Tang C Lv JC Shi SF Chen YQ Liu LJ Zhang H. Long-term safety and efficacy of hydroxychloroquine in patients with IgA nephropathy: a single-center experience. J Nephrol. 2022;35:429–40. 33591553 10.1007/s40620-021-00988-1

[93] Barratt J Lafayette R Kristensen J . Results from part A of the multi-center, double-blind, randomized, placebo-controlled NefIgArd trial, which evaluated targeted-release formulation of budesonide for the treatment of primary immunoglobulin A nephropathy. Kidney Int. 2023;103:391–402. 36270561 10.1016/j.kint.2022.09.017

[94] Lafayette R Kristensen J Stone A . Efficacy and safety of a targeted-release formulation of budesonide in patients with primary IgA nephropathy (NefIgArd): 2-year results from a randomised phase 3 trial. Lancet. 2023;402:859–70. 37591292 10.1016/S0140-6736(23)01554-4

[95] Kunter U Seikrit C Floege J. Novel agents for treating IgA nephropathy. Curr Opin Nephrol Hypertens. 2023;32:418–26. 37382182 10.1097/MNH.0000000000000902

[96] Reich HN Floege J. How I treat IgA nephropathy. Clin J Am Soc Nephrol. 2022;17:1243–6. 35675911 10.2215/CJN.02710322 PMC9435972

[97] Barbour S Feehally J. An update on the treatment of IgA nephropathy. Curr Opin Nephrol Hypertens. 2017;26:319–26. 28399021 10.1097/MNH.0000000000000336

[98] Lafayette RA Canetta PA Rovin BH . A randomized, controlled trial of rituximab in IgA nephropathy with proteinuria and renal dysfunction. J Am Soc Nephrol. 2017;28:1306–13. 27821627 10.1681/ASN.2016060640 PMC5373458

[99] Mathur M Barratt J Chacko B . A phase 2 trial of sibeprenlimab in patients with IgA nephropathy. N Engl J Med. 2024;390:20–31. 37916620 10.1056/NEJMoa2305635 PMC7615905

[100] Zhang H Rizk DV Perkovic V . Results of a randomized double-blind placebo-controlled Phase 2 study propose iptacopan as an alternative complement pathway inhibitor for IgA nephropathy. Kidney Int. 2024;105:189–99. 37914086 10.1016/j.kint.2023.09.027

[101] Caster DJ Lafayette RA. The treatment of primary IgA nephropathy: change, change, change. Am J Kidney Dis. 2024;83:229–40. 37742867 10.1053/j.ajkd.2023.08.007

